# The Neuroprotective Effects of Histamine H3 Receptor Antagonist E177 on Pilocarpine-Induced Status Epilepticus in Rats

**DOI:** 10.3390/molecules24224106

**Published:** 2019-11-14

**Authors:** Alaa Alachkar, Sheikh Azimullah, Shreesh K. Ojha, Rami Beiram, Dorota Łażewska, Katarzyna Kieć-Kononowicz, Bassem Sadek

**Affiliations:** 1 Department of Pharmacology & Therapeutics, College of Medicine and Health Sciences, United Arab Emirates University, Al Ain 17666, UAE; 2 Department of Technology and Biotechnology of Drugs, Faculty of Pharmacy, Jagiellonian University Medical College, Medyczna 9 St., 30-688 Kraków, Poland

**Keywords:** histamine H3 receptor, antagonist, pilocarpine, status epilepticus, neuroprotection, oxidative stress, rats

## Abstract

Epilepsy is a multifaceted neurological disorder which severely affects neuronal function. Some patients may experience status epilepticus (SE), a life-threatening state of ongoing seizure activity linked to cognitive dysfunction, necessitating an immediate intervention. The potential of histamine H3 receptors in several neuropsychiatric diseases including epilepsy is well recognized. In the current study, we aimed to explore the effect of H3R antagonist E177 on prevention and termination of pilocarpine (PLC)-induced SE in rats as well as evaluating the effects of E177 on the levels of oxidative stress in hippocampus tissues. The results showed that the survival rate of animals pretreated with E177 (5 and 10 mg/kg, intraperitoneal (i.p.)) was significantly increased during the first hour of observation, and animals were protected from SE incidence and showed a prolonged average of latency to the first seizure when compared with animals pretreated with PLC (400 mg/kg, i.p.). Moreover, the protective effect of E177 (10 mg/kg) on SE was partially reversed when rats were co- administered with H3R agonist *R*-(α)-methylhistamine (RAM) and with the H2R antagonist zolantidine (ZOL), but not with the H1R antagonist pyrilamine (PYR). Furthermore, pretreatment with E177 (5 and 10 mg/kg) significantly decreased the abnormal levels of malondialdehyde (MDA), and increased levels of glutathione (GSH) in the hippocampal tissues of the treated rats. However, E177 failed to modulate the levels of catalase (CAT), superoxide dismutase (SOD), or acetylcholine esterase activity (AChE). Our findings suggest that the newly developed H3R antagonist E177 provides neuroprotection in a preclinical PLC-induced SE in rats, highlighting the histaminergic system as a potential therapeutic target for the therapeutic management of SE.

## 1. Introduction

Epilepsy is a multifaceted neurological disorder which severely affects neuronal function [1,2]. Some patients may experience status epilepticus (SE), a life-threatening state of ongoing seizure activity linked to cognitive dysfunction, high mortality rate and comorbidities, necessitating immediate intervention with suitable pharmacological treatments [1,2,3,4,5]. Currently, the treatment of SE relies on immediate seizure termination using several drugs, e.g., benzodiazepines, antiepileptic drugs, and some anesthetics, which control approximately two-thirds of seizures. However, these drugs often aggravate cognitive functions [6,7,8]. Therefore, novel chemical entities effective in treating SE are still required. To investigate prospective treatments for SE in rodents, the pilocarpine (PLC)-induced SE model is usually used [9]. The PLC-induced SE exhibit behavioral and electroencephalographic features similar to those observed in human temporal lobe epilepsy (TLE) [8,10], and it proved its effectiveness as a model to investigate the pathophysiology and development of TLE [9,11]. Previous preclinical and clinical investigations have shown that there is a strong correlation between seizure pathophysiology and the brain histaminergic neurotransmission system [12,13]. Moreover, previous studies indicated that the brain histamine (HA) functions as an endogenous anticonvulsant, and intracerebroventricular (i.c.v.) injection of HA was found to prevent amygdaloid-kindled seizures in tested animals [14,15]. Furthermore, intraperitoneal (i.p.) injection of compounds that are capable of increasing the levels of brain HA (e.g., histidine and metoprine) were found not only to inhibit the amygdaloid-kindled seizures [14,15], but also to delay the length of electrically-induced seizures, and to extend the threshold of pentylenetetrazole (PTZ)-induced seizures in rodents [16]. On the other hand, brain-penetrating H1R antagonists were found to cause convulsions in diverse experimental animals models [15,17,18,19]. Interestingly, H3R antagonists/inverse agonists have gained great attention over the last decades, considering their prospective therapeutic use in the treatment of many neuropsychiatric diseases including epilepsy, Alzheimer’s disease, dementia, cognitive impairment, autism spectrum disorder, and narcolepsy [20,21,22,23,24,25,26,27]. In a recent study, the novel histamine H3R antagonist E177, which belongs to the non-imidazole histamine H3 receptor (H3R) antagonists class, was found to provide full in vivo protection against seizures in three different seizure models in rodents, including maximal electroshock- and pentylenetetrazole-induced generalized tonic-clonic seizure, following acute systemic administration (2.5, 5, 10, and 15 mg/kg, i.p.) [28]. H3Rs are coupled to Gα_i/o_-proteins and are mainly expressed in the CNS presynaptically, where they function as inhibitory auto- and hetero-receptors that modulate the biosynthesis and release of HA and other neurotransmitters such as dopamine, serotonin, acetylcholine, norepinephrine, and glutamate [22,27,29,30,31]. Consequently, H3Rs appear to be an attractive target for the design of new H3R antagonists suitable for the treatment of many neuropsychiatric diseases, including epilepsy [20,21,22,23,24,25,26,27]. On the other hand, huge attention has been given to antioxidant compounds and their role in diminishing the progression of different chronic diseases e.g., epilepsy, diabetes, cancer, autism spectrum disorder, and coronary heart disease [32,33]. Preclinical investigations revealed the role of reactive oxygen species (ROS) in PLC-induced seizure progression [34]. Also, ROS was found to be involved in the neuronal injury and neurodegeneration associated with SE in the PLC-induced seizure model [11,35]. Interestingly, several H3R antagonists showed antioxidant effect in animal models, as the H3R antagonist DL77 was found to attenuate the oxidative stress levels in the prenatal valproic acid-induced mouse model of autism [25]. Based on the aforementioned experimental findings and as a continuation of our work, the protective effects of the potent non-imidazole H3R antagonist/inverse agonist, namely E177 [1-(6-(naphthalen-2-yloxy)hexyl)azepane], with high antagonist affinity (*K*_i_ = 69.40 nM) [36,37] and high in vitro selectivity towards H3Rs, were assessed in the PLC-induced SE model. Moreover, the effects of E177 on oxidative stress markers as well as AChE activity in the hippocampus of tested animals were examined. In addition, abrogative studies were carried out by co-administering CNS penetrant H1R antagonist pyrilamine (PYR), H2R antagonist zolantidine (ZOL), and H3R agonist (*R*)-α-methyl histamine (RAM) to test whether brain histaminergic neurotransmissions are involved in the effects provided by the H3R antagonist E177. The H3R antagonist E177 was selected for the current series of experiments, as previous experiments indicated its promising anticonvulsant effect in different acute seizure models, e.g., maximal electroshock (MES) and PTZ-induced seizure model [23,30,38,39]. Moreover, E177 has recently shown a dose-dependent procognitive effect in dizocilpine-induced amnesia in rodents without modulation of anxiety-like behaviors [37].

## 2. Results

### 2.1. E177 Prolonged Latency Time to First Seizure

**Figure 1:**
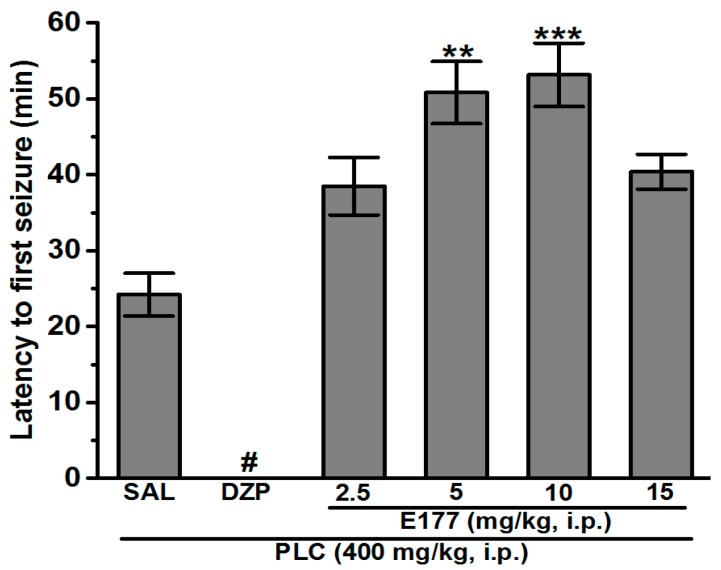
Effects of pretreatment with DZP and H3R antagonist E177 on latency time to PLC-induced seizure. Effects of acute systemic injection with E177 (2.5, 5, 10, and 15 mg/kg, i.p.) or DZP (10 mg/kg, i.p.) on latency time (min) to the first PLC-induced seizure. ** *p* < 0.01 as compared with PLC group, *** *p* < 0.001 as compared with PLC group, ^#^ Full protection as compared to PLC group. Results are expressed as mean ± S.E.M (*n* = 8).

All seizures observed were generalized tonic-clonic convulsions with SE. Observation of the rats for 1 h following systemic administration of PLC (400 mg/kg i.p.) showed that the average of the latency time to the first seizure was 24.20 ± 2.82 min, while no seizures were observed in animals pretreated with the reference drug diazepam (DZP, 10 mg/kg i.p.) (*p* < 0.001) (Figure 1). Moreover, the results showed that pretreatment with E177 (2.5 and 15 mg/kg i.p.) failed to significantly prolong the average of the latency to first seizure with 38.5 ± 3.78 and 40.40 ± 2.30 min, respectively, and as compared to the PLC group. However, pretreatment with E177 (5 and 10 mg/kg i.p.) significantly extended the average of the latency time to the first seizure with 50.83 ± 4.1 and 53.17 ± 4.17 min, respectively, and as compared to the PLC group (all *p* < 0.01) (Figure 1).

### 2.2. Effect of E177 on SE Incidence and Survival Rate

The observed results revealed that all tested animals injected with PLC (400 mg/kg, i.p.) showed convulsions with SE and 66.66% of the animals survived from SE after 1 h from injection (Table 1). However, test animals pretreated with DZP (10 mg/kg, i.p.) did not experience convulsions and survived. In addition, the observed results showed that pretreatment with E177 (2.5, 5 and 15 mg/kg, i.p.) showed tendency to reduce the average of SE incidence to 50% in comparison to the PLC group, while E177 (10 mg/kg) reduced the average of SE incidence to 33.33% in comparison to the PLC group. However, it was observed that survival rate was increased to 100.00% with all E177 doses except for E177 (15 mg/kg) which raised the survival rate only to 83.33% (Table 1). Performed analyses applying the Chi-square test (nonparametric test (X2)) indicated that DZP treatment 30–45 min before PLC treatment significantly reduced both SE incidence and survival rate when compared with the PLC- treated group with (*p* < 0.001 and *p* < 0.05), respectively (Table 1). Moreover, all doses of E177 (2.5, 5, 10 and 15 mg/kg, i.p.) significantly reduced SE incidence when compared with the PLC-treated group (all *p* < 0.05) except for E177 10 mg/kg (*p* < 0.001). Furthermore, E177 (2.5, 5, and 10 mg/kg, i.p.) significantly increased survival rate when compared with the PLC-treated group (all *p* < 0.05). However, E177 15 (mg/kg, i.p.) failed to significantly raise the survival rate when compared with the PLC-treated group (Table 1).

### 2.3. Effects of RAM, PYR, and ZOL on E177-Provided Protection against SE

**Figure 2:**
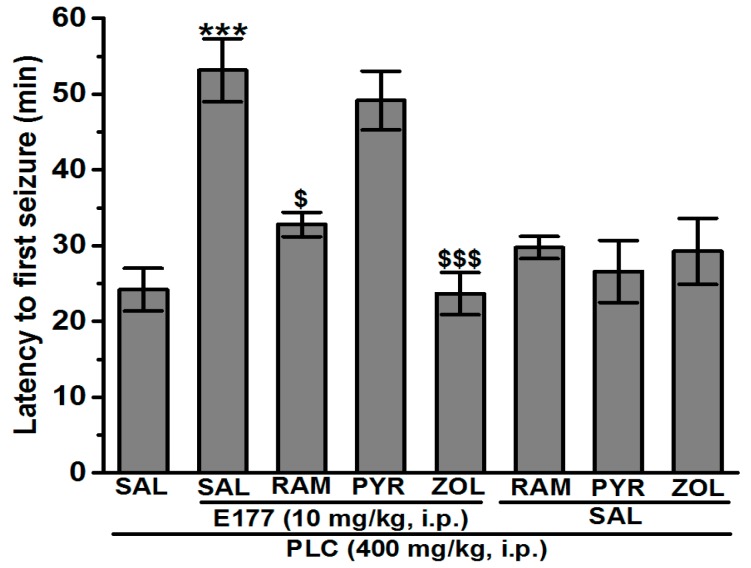
Effect of H3R agonist RAM, H1R antagonist PYR, and H2R antagonist ZOL on E177- provided prolongation of latency time in PLC-induced seizure. Effects of acute systemic injection with E177 (10 mg/kg, i.p.), E177(10 mg/kg) + RAM(10 mg/kg), E177(10 mg/kg) + PYR(10 mg/kg), and E177(10 mg/kg) + ZOL(10 mg/kg) on latency time (min) to the first PLC-induced seizure. *** *p* < 0.001 as compared with PLC group, ^$^ *p* < 0.05 as compared with E177 (10 mg/kg) group, ^$$$^ *p* < 0.001 as compared with E177 (10 mg/kg) group. Results are expressed as mean ± S.E.M (*n* = 8).

The abrogation of the most promising protective dose of E177 (10 mg/kg, i.p.) was evaluated by systemic co-injection of RAM (10 mg/kg i.p.), PYR (10 mg/kg i.p.), and ZOL (10 mg/kg i.p.) before PLC injection (Figure 2). The results showed that PYR (10 mg/kg i.p.) co-administration failed to significantly abrogate the average of the latency to first seizure and SE incidence average in comparison to animals treated with E177 (10 mg/kg) (*p* > 0.05). However, co-injection with ZOL (10 mg/kg i.p.) significantly reversed the protective effects of E177 (10 mg/kg) on the average of latency to first seizure (23.66 ± 2.79 min, *p* < 0.001). Also, co-injection with ZOL partially reversed the SE incidence average to 100% (*p* < 0.05) and reduced the survival rate to 83.33% (*p* < 0.05) (Figure 2 and Table 1). Moreover, co-injection with RAM (10 mg/kg i.p.) significantly decreased the average of the latency to first seizure to 32.80 ± 1.45 min (*p* < 0.05) (Figure 2), and raised the SE incidence average to 66.67% (Table 1), and no change was observed on the level of survival rate (Table 1). Notably, systemic administration of RAM, PYR, and ZOL alone did not alter the average of the latency to first seizure and SE incidence average in comparison to PLC group (*p* > 0.05) (Figure 2).

### 2.4. Effects of E177 on Oxidative Stress Markers

**Figure 3:**
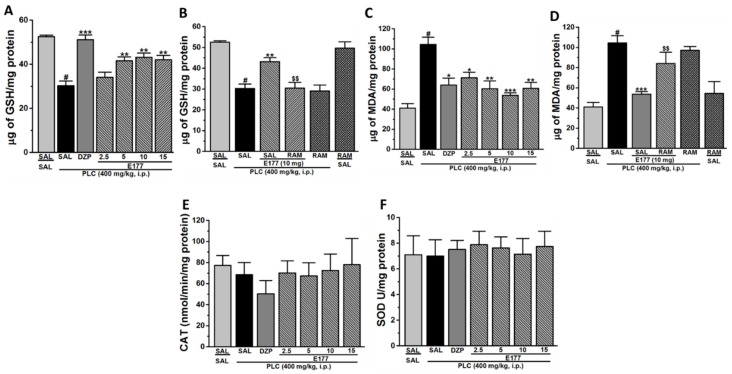
Effects of E177 on status of oxidative stress levels in rat hippocampus after 1 h of PLC-induced seizures. Modulated levels of glutathione (GSH), malondialdehyde (MDA), catalase (CAT), and superoxide dismutase (SOD) were assessed. PLC-(400 mg)-treated mice showed a significant decrease in GSH levels (**A**) and a significant increase in MDA (**C**), but failed to alter CAT (**E**) or and SOD (**F**) levels compared with saline-treated rats. E177 (2.5, 5, 10, or 15 mg/kg i.p.) or DZP (10 mg/kg, i.p.) were administered 30–45 min prior to PLC-injection. E177 (5, 10, and 15 mg/kg, i.p.) or DZP (10 mg/kg, i.p.) significantly mitigated GSH levels (**A**) and MDA levels (**C**). Effects of systemic co-injection with RAM (10 mg/kg, i.p.) on E177 (10 mg)-provided modulation of oxidative stress levels were assessed (**B&D**). ^#^ *p* < 0.001 as compared to Saline-treated rats, * *p* < 0.05, ** *p* < 0.01 or *** *p* < 0.001 as compared to PLC-treated rats. ^$$^ *p* < 0.01 as compared to E177(10 mg/kg) group. Results are expressed as mean ± S.E.M (*n* = 8).

The results indicated that malondialdehyde (MDA) levels were significantly increased in the PLC group when compared to the control group treated with saline (*p* < 0.001), while glutathione (GSH) levels were markedly decreased (*p* < 0.0001), and no significant difference was observed of both catalase (CAT) and superoxide dismutase (SOD) levels (Figure 3E,F). Moreover, the observed results showed that glutathione (GSH) levels were noticeably elevated in the DZP group (*p* < 0.001) as compared to the PLC group, also MDA levels were significantly reduced in the DZP group (*p* < 0.05) as compared to PLC group (Figure 3A,C). Furthermore, the results showed that pre-treatment with E177 (2.5, 5, 10 and 15 mg/kg, i.p.) significantly reduced MDA levels (all *p* < 0.05) when compared to PLC group. In addition, the results demonstrated that pretreatment with E177 (5, 10 and 15 mg/kg, i.p.) markedly increased GSH levels (all *p* < 0.01) in comparison to the PLC group, while unnoticeable change was observed in animals treated with the lower dose of E177 (2.5 mg/kg, i.p.) (Figure 3A). Interestingly, the results indicated that pretreatment with RAM (10 mg/kg i.p.) significantly abrogated the protective effect observed by E177 (10 mg/kg i.p.) on the levels of MDA and GSH (all *p* < 0.01) (Figure 3B,D). Notably, RAM administered alone with PLC or with SAL did not modulate the levels of MDA and GSH in comparison to PLC group as well as SAL group (all *p* > 0.05).

### 2.5. Effects of E177 on AChE Activity

**Figure 4:**
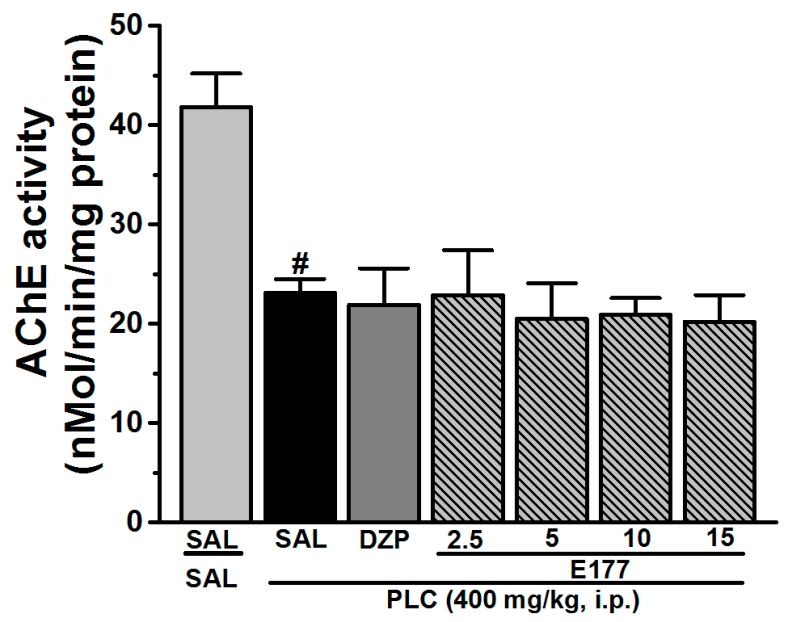
Effects of E177 on AChE activity in rat hippocampus tissues of PLC-seizure rats. Effects of acute systemic injection of rats with E177 (2.5, 5, 10, and 15 mg/kg, i.p.) or DZP (10 mg/kg, i.p.) on acetylcholine esterase enzyme in the hippocampus of PLC-seizure rats. Quantitative analysis revealed a significant decrease (^#^ *p* < 0.001) in the AChE activity in the hippocampus of PLC-seizure rats compared to the control rats. Acute systemic treatment with E177 (2.5, 5, 10, and 15 mg/kg, i.p.) or DZP (10 mg/kg, i.p.) to the PLC-rats failed to restore this activity compared to the PLC-treated rats. Values are expressed as the percent mean ± SEM (*n* = 6–8).

The results showed that acetylcholine esterase (AChE) activity was significantly decreased in the PLC group (*p* < 0.001) when compared to the control group (Figure 4). However, no significant change was observed in animals pretreated with E177 (2.5, 5, 10, and 15 mg/kg, i.p.) (Figure 4).

## 3. Discussion

Early pathophysiological insults of SE in rodents consist of intense neuroinflammation, imbalanced oxidative stress, selective neuronal degeneration, and disruption of the brain-blood barrier, which result in subsequent cognitive deficits [40,41]. H3Rs are considered a promising potential target for treating different brain disorders e.g., epilepsy, autism spectrum disorder, Alzheimer’s disease, dementia, cognitive impairment, and narcolepsy [20,21,22,23,24,25,26,27]. Moreover, numerous H3R antagonists were previously investigated on their protective effects in different acute seizure models, and the results revealed their anticonvulsant effects [23,30,42,43,44,45,46,47]. In this study and as a continuation of our previous work, the protective effect of the non-imidazole based H3R antagonist E177 with high in vitro antagonist affinity (*h*H3R *K*_i_ = 69.3 nM) [36,37] and excellent selectivity profile [28] towards H3Rs was investigated in the PLC-induced SE model. Supported by the behavioral and morphological outcomes as well as electroencephalographic waves observed in previous preclinical studies, the PLC-induced seizure model has been proposed as an animal model with similarity in characteristics to TLE, and numerous previous studies showed that acute systemic administration of a high dose of the muscarinic cholinergic agonist PLC (400 mg/kg) provoked behavioral alternations and convulsions in all tested animals which developed within 30 min to SE [8,9,11,48,49,50]. Our observations in the present study agreed with these previous studies. Interestingly, systemic pretreatment with the reference drug DZP (10 mg/kg, i.p.), a traditional antiepileptic drug (Figure 1), prevented the seizures and SE occurrence induced by PLC, and this result was also in line with previous experimental observations [51]. In addition, our results indicated that pretreatment with E177 (5 and 10 mg/kg) significantly prolonged the average of the latency to the first seizure, reduced the average seizure and SE incidence, and increased the survival rate after SE (Figure 1, Table 1). However, no protective effect was observed following pretreatment with E177 (2.5 and 15 mg/kg), indicating a dose-dependent effect of E177 in PLC-induced seizure model. The latter dose-dependent effect of E177 is in agreement with previous preclinical outcomes that demonstrated a dose-dependent effect of different H3R antagonists in several animal models [23,25,30], and with our previous observation of a dose-dependent anticonvulsant effect of E177 in electrical as well as chemically induced seizure models [28]. Importantly, the protection provided by E177 (5 and 10 mg/kg) in the PLC-induced seizure model was significantly higher when compared to the higher dose (15 mg/kg, i.p.) or the lower dose (2.5 mg/kg, i.p.), demonstrating that an optimum in protective effect was observed when the H3R antagonist E177 was applied at the dose of 5 or 10 mg/kg, and an off-target effect for E177 at higher doses (15 mg/kg). However, insufficient antagonistic interactions of the test compound E177 with H3Rs might have been observed with the lower dose (2.5 mg/kg) of E177(Figure 1). The latter observations of dose dependency are, also, in agreement with earlier experimental results conducted in different rodents [24,37,52,53,54,55]. Moreover, the E177-provided protection was significantly reversed when animals were administered with the centrally acting H3R agonist RAM (10 mg/kg) or the centrally acting H2R antagonist ZOL (10 mg/kg), but not with the centrally acting H1R antagonist PYR, suggesting that the protective effect of E177 is mediated through modulation of the central histaminergic neurotransmission and to some extent with interaction of the released HA with post-synaptically located H2Rs but not H1Rs (Figure 2).

Oxidative stress has been found to significantly contribute in the initiation and progression of epilepsy [56,57], and previous studies revealed that PLC-induced SE led to elevated oxidative stress, and therefore, to increased levels of brain ROS and neuronal damage [50,58,59,60,61,62]. Therefore, measurement of several oxidative stress markers (e.g., MDA, GSH, CAT, and SOD) of tested animals was necessary to evaluate the mitigating effects of a prospective novel and centrally acting compound like E177. Moreover, and among all the brain regions, the hippocampus has gained the attention in the SE model as it includes numerous definite neuronal circuits connected to seizure genesis in addition to the fact that the hippocampus is particularly susceptible to PLC-induced neuronal injury. Our observed results indicated an elevated MDA level accompanied by a reduction in GSH levels in the hippocampus 1 h post PLC-induced seizures, and these results were in agreement with previous studies conducted on several rodents [9,63,64,65,66]. On the other hand, natural antioxidant enzymes in the brain, e.g., CAT and SOD, are typically used to scavenge the elevated ROS and defend the hippocampus from convulsions. However, numerous previous studies revealed that the level of CAT was decreased after 6 h following seizure initiation with no change in SOD levels [61,66], whereas others showed low levels of hippocampal CAT and SOD 24 h following seizure [34,48,64]. Our observations revealed no change in the hippocampal levels of CAT and SOD activities 1 h after seizures, suggesting that an increased metabolic demand can be indicated during the epileptic seizure, and CAT and SOD activities are not alerted in this acute phase of PLC-induced seizure (Figure 3E,F). Interestingly, systemic pretreatment with different doses of E177 attenuated the increased oxidative stress caused by PLC-induced seizures, as E177 (5, 10, and 15 mg/kg) rescued the depletion of GSH levels (Figure 3A) and reduced the elevated levels of MDA (Figure 3C) in the hippocampus of tested animals, demonstrating the antioxidant potential of H3R antagonist E177. However, the observed antioxidant effect for E177 was found to be independent of the dose administered, as 5, 10, and 15 mg/kg were statistically not different in providing protective effects (Figure 3A,C). The latter observation of dose independency is in line with previous observations in which the H3R antagonist DL77 (10 or 15 mg/kg, i.p.) provided comparable antioxidant effects against increased oxidative stress parameters in the hippocampus and cerebellum of valproic acid- induced autism-like behaviors in mice [25]. Interestingly, the E177-provided effects on the levels of GSH and MDA were completely reversed when animals were co-administered with the CNS-penetrant H3R agonist RAM, indicating the involvement of brain histaminergic H3R neurotransmission in the provided mitigating effects of E177 on the levels of GSH as well as MDA (Figure 3B,D). The latter results are in agreement with a recent study in which H3R antagonist DL77 mitigated the oxidative stress by reducing MDA and increasing GSH levels in a valproic acid-induced autism-like paradigm in mice [25].

The involvement of cholinergic neurotransmission in the PLC-induced seizure model has been well documented [8,50]. PLC-induced seizures were associated with an increase in cholinergic activity in the hippocampus of tested animals due to neuronal hyperactivity [67], which might lead to neuronal damage during seizures and SE induced by PLC [68]. Moreover, the memory impairment associated with the PLC-induced seizure model has previously been correlated with changes in functionality of the brain cholinergic system [69]. Also, it is well known that impaired function of AChE, an enzyme that catalyzes the breakdown of acetylcholine, is accompanied by memory impairment [70], since AChE has a significant part in cholinergic neurotransmission [71], and low levels of AChE usually result in abnormal elevated levels of the neurotransmitter acetylcholine in the cholinergic synapses, which lead to excessive motivation and stimulation of muscarinic and nicotinic receptors [72]. Therefore, it was important to assess the levels of AChE in the hippocampus of the tested animals following PLC-induced seizures and SE. Our observed results revealed a significant decrease in the AChE activity in the hippocampus 1 h after systemic injection of PLC, demonstrating a possible increase in the brain levels of acetylcholine. Also, previous preclinical studies showed that PLC systemic administration altered AChE activity, suggesting an essential correlation of AChE activity in the hippocampus and the pathogenesis of PLC-induced SE [73]. Notably, systemic pretreatment with E177 failed to provide any significant alternation of AChE levels in the hippocampus of treated animals (Figure 4).

## 4. Material and Methods

### 4.1. Animals

Inbred male Wistar rats (body weight 180–220 g) used in this current study were obtained from the central animal facility in UAE University. The animals were kept in a specific room with controlled temperature and humidity (24 °C ± 2 °C and 55% ± 15%, respectively), 12/12 h light/dark sequence, and free access to food and water. The in vivo experiments were performed each day between 09.00 and 13.00 by the same investigator in a blinded manner. All procedures were performed in accordance with the guidelines of the European Communities Council Directive of 24 November 1986 (86/609/EEC) and were previously approved for epilepsy study by the College of Medicine and Health Sciences/United Arab Emirates University (Institutional Animal Ethics Committee, approval number; ERA_2017_5676). All efforts were made to minimize the total number of animals used and to diminish the suffering of the animals.

### 4.2. Drugs

The H3R antagonist E177 was synthesized in the Department of Technology and Biotechnology of Drugs (Jagiellonian University Medical College, Kraków, Poland), as previously described [36]. Diazepam (DZP) was produced by Gulf Pharmaceutical Industries (Ras Al Khaimah, United Arab Emirates) and was acquired from Dr. Ameen Al Amaydah (Department of Emergency Medicine, Emirates International Hospital, Al Ain, and United Arab Emirates). The histamine H3R agonist *R*-(α)-methylhistamine dihydrochloride (RAM), pyrilamine maleate (PYR), zolantidine dimaleate (ZOL), pilocarpine hydrochloride (PLC), and scopolamine methyl nitrate were obtained from Sigma-Aldrich (St Louis, MO, USA). All compounds were dissolved in isotonic saline and administered intraperitoneal (i.p.) at a volume of 1 mL/kg. All doses of test compounds were expressed in terms of the free base and were determined by previous studies [24,28,37,39,74].

### 4.3. Experimental Procedure of PLC-Induced SE

**Figure 5:**
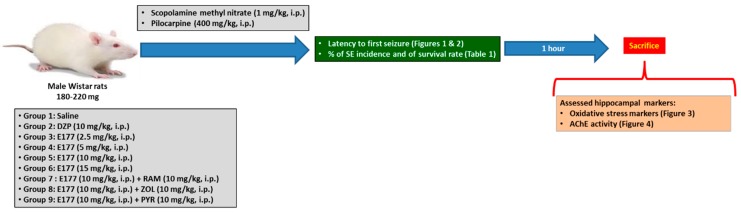
Schematic diagram of drug treatments, seizure studies, and biochemical assessments with rats.

In a set of experiments, animals were divided into 7 groups of 8–12 animals as follows; group 1: control group (saline 0.9% i.p.), group 2: pilocarpine group (PLC 400 mg/kg i.p.), group 3: positive control group (PLC 400 + DZP 10 mg/kg i.p.), group 4, 5, 6, and 7: treatment groups (PLC 400 + E177 2.5, 5, 10 or 15 mg/kg i.p.) (Figure 5). All groups were treated with scopolamine methyl nitrate (1 mg/kg i.p.) 30 min before PLC injection to reduce the peripheral cholinergic effects of PLC. All treatments (DZP and E177) were given 30–45 min before PLC injection. After the PLC administration all the animals were placed in separate cages to observe their behavior, record the latency to first seizure (any epileptic behavioral viewed after PLC administration, e.g., wild running, clonus, tonus, and tonic-clonic seizures), number of animals that experienced SE and rate of survival at 1 h after injection of PLC. Previous studies indicated that seizures and deaths were observed within 1 and 24 h after PLC injection constantly pursued in a similar manner [65,75]. In the present study, convulsions occurred within 30–60 min and deaths within 1 h post PLC injection in tested rats, and the PLC group consisted of the rats which experienced SE and survived within 1 h. In addition, abrogation studies with the most promising protective dose of E177 were carried out in three additional groups for further analyses. Consequently, all animals received the most protective dose of E177 (10 mg/kg, i.p.) together with the H3R agonist RAM (10 mg/kg i.p.), H1R antagonist PYR (10 mg/kg i.p.), or H2R antagonist ZOL (10 mg/kg i.p.) 30–45 min before PLC injection (except for the CNS penetrant H3R agonist RAM which was administered 15–20 min before the start of tests to ensure its presence in the CNS, as RAM was described to show fast metabolism [76]). All doses were selected according to previous studies [24,28,37,39,74].

### 4.4. Biochemical Assessments

All rats (*n* = 8) that survived within 1 h after PLC injection were anesthetized with pentobarbital (40 mg/kg body weight); cardiac perfusion was carried out to flush the blood out using 0.01 M phosphate-buffered saline (PBS). The brains were quickly placed on an ice-plate and dissected in order to remove the hippocampus which was immediately frozen in liquid nitrogen for further future use.

#### 4.4.1. Oxidative Stress

The hippocampus was homogenized in KCL buffer (Tris-HCl, 10 mM NaCl, 140 mM KCl, 300 mM EDTA, 1 mM Triton-X-100 0.5%) at pH 8.0 supplemented with protease and phosphatase inhibitor. Then the mixture was centrifuged at 10,000× *g* for 30 min at 4 °C. Subsequently, the supernatant was collected to be used in the assessment of oxidative stress markers as described previously [25,77,78]

##### 4.4.1.1. MDA

Malondialdehyde (MDA) is an index of lipid peroxidation. MDA was estimated using (North West Life Science, Vancouver, WA, USA) detection kit. The samples and standards were incubated with butylated hydroxytoluene, and 2-thiobarbituric acid for 60 min at 60 °C. Subsequently, all the samples and standards were centrifuged for 2–3 min at 10,000× *g*, followed by assessment of the absorbance at 532 nm. The level of MDA was expressed as μM MDA/mg protein.

##### 4.4.1.2. GSH

Reduced glutathione (GSH) is the major free thiol in most living cells and acts as an antioxidant. GSH levels were measured using (Sigma-Aldrich, Sanit Louis, MO, USA) detection kit. The samples were deproteinized by adding 5-sulfosalicylic acid solution and centrifuging the samples for 10 min at 10,000× *g* to remove any precipitated protein, and the supernatant was collected to measure the levels of GSH. In 96-well plates, the samples and standards were incubated for 5 min at room temperature (RT) with the working reagent (glutathione reductase, 5,5-dithiobis (2-nitrobenzoic acid) and assay buffer). Subsequently, the samples and standards were diluted with NADPH solution. Finally, the absorbance of the samples and standards was measured at 412 nm each minute for 5 min using the micro plate reader. The level of GSH was expressed as μM GSH/mg protein.

##### 4.4.1.3. CAT

Catalase (CAT) is an antioxidant enzyme that is found in all aerobic organisms. CAT levels were quantified using the Cayman Chemical Company, Ann Arbor, MI, USA detection kit. In 96-well plates all samples and standards were mixed with assay buffer and methanol. The reaction was started by adding H_2_O_2_ to each well, then the 96-well plate was covered and incubated for 20 min at room temperature on a shaker. Potassium hydroxide was added in order to stop the reaction, followed by the addition of catalase purpald to each well again, and the plate was covered and incubated for 10 min at room temperature on the shaker. Potassium periodate was added and incubation for 5 min was carried out. Finally, absorbance was recorded at 540 nm using a micro plate reader. CAT activity was expressed as the amount of enzyme required for the formation of 1 nmol of formaldehyde per min (nmol/min/mL).

##### 4.4.1.4. SOD

Superoxide dismutase (SOD) is an important antioxidant enzyme defense in all living organisms. SOD levels were measured using the Cayman Chemical Company, Ann Arbor, MI, USA detection kit. First, all samples and standards were added to radical detector in a 96-well plate, then xanthine oxidase was added to each well to initiate the reaction. The plate was covered and incubated for 30 min at room temperature on a shaker. Finally, absorbance was recorded at 450 nm using a micro plate reader. SOD activity was expressed as (U/mL), where each unit represents the amount of the enzyme required to reveal 50% dismutation of the superoxide radical.

##### 4.4.1.5. Acetylcholine Esterase (AChE) Activity

The hippocampus was homogenized in phosphate buffer (PB) at pH 8.0. Then the mixture was centrifuged at 10,000× *g* for 30 min at 4 °C and the supernatant was collected to be used in the assessment of AChE. The level AChE was measured using (Biovision company, Milpitas, CA, USA) colorimetric assay kit. All samples were diluted in 96-well plate by AChE assay buffer, then the reaction mix (AChE assay buffer, AChE probe, and AChE substrate and choline oxidase enzyme mix) were added to initiate the reaction in each well. Afterward, the plate was covered and incubated for 20–30 min at 37 °C. Finally, the absorbance was recorded at 570 nm using a micro plate reader.

### 4.5. Statistical Analysis

For statistical comparisons, the software package SPSS 25.0 (IBM Middle East, Dubai, and UAE) was used. All data are expressed as the means ± standard error of mean (SEM). Protective effects observed for H3R antagonist E177 in PLC-induced seizure Model were analyzed using one-way analysis of variance, followed by the Bonferroni post hoc test for multiple comparisons. The results observed for percentage seizures and survival are expressed as percentages of the number of animals from each experimental group and were analyzed using a nonparametric test (X^2^). The criterion for statistical significance was set at *p* < 0.05.

## 5. Conclusions

Taken together, the results of the present study demonstrate that the novel non-imidazole-based H3R antagonist E177 prevented PLC-induced convulsions, SE, and mortality in the tested animals. Moreover, the E177-provided protection was partially reversed with the CNS-penetrant RAM and ZOL, indicating a correlation between the brain histaminergic neurotransmission and the protective effects provided by E177. Furthermore, the observed results suggest that oxidative stress is significantly involved in the development and/or maintenance of seizure activity, and E177 significantly reduced the susceptibility of tested animals to PLC-induced seizure. However, a battery of additional seizure test models with different species is still warranted to further comprehend the current results observed for H3R antagonist E177, and to strengthen the translational value of its prospective applicability in the therapeutic management of SE.

## Figures and Tables

**Table 1:** Effects of pretreatment with DZP and H3R antagonist E177 after pilocarpine-induced seizures.

Group	Percentage of SE Incidence (%)	Percentage of Survival (%)
SAL	0.00	100.00
PLC (400 mg/kg)	100.00 ^#^	66.67
DZP (10 mg/kg)	0.00 **	100.00
E177 (2.5 mg/kg)	50.00 *	100.00
E177 (5 mg/kg)	50.00 *	100.00
E177 (10 mg/kg) ^a^	33.33 **	100.00
E177 (15 mg/kg)	50.00*	83.33
E177 ^a^ + RAM ^b^	66.67	100.00
E177 ^a^ + PYR ^c^	50.00	100.00
E177 ^a^ + ZOL ^d^	100.00 ^$^	83.33
RAM + PLC	83.33	100.00
PYR + PLC	83.33	83.33
ZOL + PLC	66.67	100.00

^a^ (10 mg/kg i.p.), ^b^ (10 mg/kg i.p.), ^c^ PYR (10 mg/kg i.p.), ^d^ ZOL (10 mg/kg i.p.). ^#^ *p* < 0.001 as compared to Saline-treated rats, * *p* < 0.05, ** *p* < 0.001 as compared to PLC-treated rats. ^$^ *p* < 0.05 as compared E177(10 mg/kg)-treated group. Values are expressed as percentages of the number of animals from each experimental group (*n* = 12).
